# Irregularity of the Teeth

**Published:** 1844-06-01

**Authors:** J. F. B. Flagg

**Affiliations:** Surgeon Dentist; Philadelphia


### IRREGULARITY OF THE TEETH.

To the Editor of the Medical Examiner.

Dear Sir,—Deformity or irregularity of the permanent set of teeth is a subject but little understood, in proportion to the frequency of its occurrence at the present day, except by the scientific dentist. The most ordinary causes of this malformation should not only be fully comprehended by the practising physician, but I conceive it to be seriously incumbent upon him to correct one or two important errors which prevail always within the circle of his practice, to the continuance of this evil.

It is my object, in this place, to ask the attention of your readers to such situations of the temporary set, or shedding teeth, as is most apt to produce this difficulty in those which are to follow, either from a neglect to remove them sufficiently early, or from too great officiousness in this matter.

If the primary tooth remains firm, after the indication of its successor, in an improper position, such tooth, and sometimes one immediately adjoining, should be removed, and a daily pressure made upon the tooth or prominence caused by its presence. As few teeth, under these circumstances, should be extracted, as may suffice to give place to the new one; as, for instance, when this difficulty occurs with an upper central incisor, in consequence of its being so much larger than its predecessor, it is not unusual to remove two teeth for its accommodation; whereas, a lateral incisor and the inferior front teeth vary so little in size in both sets, as seldom to require the removal of more than *one* for this purpose.

Another most frequent cause of deformity, to a certain extent, is the removal of the deciduous teeth entirely too early, simply, because they are discovered to be loose. Now the fact of a tooth becoming loose, implies that nature is at work most harmoniously, and ought not to be disturbed. As the upper portion of the new tooth (supposing it to be in the lower jaw) presses upon the root of the old one, it causes an absorption of that root, and, in most cases, the temporary tooth will remain in, growing more and more loose for several weeks; and finally the child removes it with his finger, or perhaps with no other agent than his tongue. When such is the case, a beautiful and true set of teeth is almost always the result. If a tooth is removed untimely in this view of the case, the arch being broken, the teeth incline toward each other, and the jaw itself contracts, and becomes too small to accommodate the full compliment of teeth which are soon to make their appearance.

In connection with the foregoing remarks, I am induced to communicate the following case, and its method of treatment; not so much from its rareness of occurrence, as its *extent* in the present instance, implicating the well-being, not only of the two central upper incisors, but those of the four inferior teeth of the same name. I would further add that I simply claim originality for so constructing my apparatus as to remedy both evils at one and the same time.

On the 28th of April last, a lad aged 12 years, son of one of our most respectable merchants, was presented to me by my friend, Dr. J. M. Wallace, under the following circumstances:

The two central upper incisors were caught behind the four front lower teeth, in such manner as that, when the jaws were closed, they were in consequence entirely concealed; this condition of the teeth not only had a tendency to force the upper ones, so situated, farther into the mouth at every effort of mastication, but also to protrude the lower far beyond their natural arch, carrying with them the lower lip, much to the inconvenience and discomfort of the patient.

Having taken an entire cast of both sets of teeth, I discovered that to use the band and blocks, as described by Joseph Fox, would force the upper set entirely too far forward if they were made to overlap the under ones in their present condition; and if the more modern invention of fitting a metallic frame to the entire lower set, fastening it by ligatures to the back, or *molar* teeth, was applied in this case, it could only have a like tendency with the former plan. It was necessary, therefore, that the instrument used for this purpose should embrace merely the teeth at fault. I had only to take a cast of the four lower incisors, (the *cuspidati* not having made their appearance above the gum;) upon these I constructed my frame-work, soldered to the outer portion of which were two *inclined planes*, riding over the tops of these teeth sufficiently high and inclined inwards to strike upon the cutting edges of the irregular teeth at every attempt to close the mouth. This caused an outward pressure to the upper, and a contrary force to the under, teeth.

In conclusion, I am happy to add that it has been worn, with perfect comfort to the patient, for three weeks, in which time an entire remedy of this unseemly defect has been attained.

J. F. B. Flagg, M. D.,
Surgeon Dentist.

*Philadelphia*, May 25th, 1844.

